# Internal Integrated Temperature Sensor for Lithium-Ion Batteries

**DOI:** 10.3390/s25020511

**Published:** 2025-01-17

**Authors:** Pengfei Yang, Kai Su, Shijie Weng, Jiang Han, Qian Zhang, Zhiqiang Li, Xiaoli Peng, Yong Xiang

**Affiliations:** 1 School of Materials and Energy, University of Electronic Science and Technology of China, Chengdu 611731, China; 2 Zhuhai China Eagle Electronic Circuit Co., Ltd., Zhuhai 519000, China; 3 Zhuhai Henger Microelectronic Equipment Co., Ltd., 6 Jinyuan First Road, Tangjiawan Town, High-Tech Zone, Zhuhai 519085, China; 4 Frontier Center of Energy Distribution and Integration, Tianfu Jiangxi Lab, Huoju Avenue, Futian Sub-District, Chengdu 641419, China

**Keywords:** internal integrated, flexible printed circuit, temperature sensor, lithium-ion battery

## Abstract

Lithium-ion batteries represent a significant component of the field of energy storage, with a diverse range of applications in consumer electronics, portable devices, and numerous other fields. In view of the growing concerns about the safety of batteries, it is of the utmost importance to develop a sensor that is capable of accurately monitoring the internal temperature of lithium-ion batteries. External sensors are subject to the necessity for additional space and ancillary equipment. Moreover, external sensors cannot accurately measure internal battery temperature due to packaging material interference, causing a temperature discrepancy between the interior and surface. Consequently, this study presents an integrated temperature sensor within the battery, based on PT1000 resistance temperature detector (RTD). The sensor is integrated with the anode via a flexible printed circuit (FPC), simplifying the assembly process. The PT1000 RTD microsensor’s temperature is linearly related to resistance (R = 3.71T + 1003.86). It measures about 15 °C temperature difference inside/outside the battery. On short-circuit, the battery’s internal temperature rises to 27 °C in 10 s and 32 °C in 20 s, measured by the sensor. A battery with the PT1000 sensor retains 89.8% capacity under 2 C, similar to the normal battery. Furthermore, a PT1000 temperature array sensor was designed and employed to enable precise monitoring and localization of internal temperature variations.

## 1. Introduction

Lithium-ion batteries (LIBs) exhibit a number of noteworthy characteristics, including high energy density, long cycle life, high power density, and low static self-discharge. Lithium-ion batteries are employed in a multitude of applications, including hybrid electric vehicles, consumer electronics, portable devices, and the field of energy storage [1,2]. However, the safety of LIBs is becoming increasingly paramount. They constitute highly complex closed electrochemical systems, and their safety is closely related to raw materials, manufacturing processes, working environments, and other factors [3,4,5]. When batteries are overcharged, aged, subjected to mechanical crush, or used inappropriately, LIBs may experience thermal runaway, leading to serious safety issues [6,7]. The occurrence of incidents involving the spontaneous combustion of cell phone batteries and the explosion of electric vehicles has caused significant harm, prompting researchers to prioritize the safety of batteries and the development of warning devices [8,9,10]. Efforts to monitor and evaluate battery states (e.g., State of Charge (SOC), State of Health (SOH), etc.) have led to numerous achievements. Various sensors have been developed to monitor voltage [11], temperature [12,13,14], current [15], stress [16], leakage [17], gas emissions [18,19], and other battery indicators. Additionally, battery safety management systems [20,21] have been constructed to ensure that batteries operate within safe limits.

Temperature is a key factor that indicates the safe operating condition of a battery [22]. Currently, employing external sensors for battery health management and analysis is a well-established approach. Commonly utilized external sensors include thermocouple (TC) sensors [23], infrared thermal imaging systems [24], resistance temperature detector (RTD) sensors [25], and fiber Bragg grating (FBG) sensors [26]. Peng et al. proposed a fiber optic sensor consisting of a metal ring and FBG for monitoring LIB temperature [27]. Hu et al. prepared a novel LIB health monitoring sensor array using piezoelectric/thermoelectric polyvinylidene fluoride-trifluoroethylene (PVDF-TrFE), enabling direct real-time response and simultaneous localization of dynamic mechanical and thermal damage [28]. Zhu et al. designed one end of a LIB sealed with BaF_2_ optical glass, allowing an infrared camera to monitor the internal temperature during charging and discharging in real time through this optical glass [29]. External sensors increase battery size and have the disadvantage of low monitoring accuracy and hysteresis due to heat conduction [30,31]. Furthermore, safety issues often arise from thermal runaway within the battery, resulting in sudden temperature changes [10,32]. Hence, to enhance battery safety monitoring and management, the implantation of sensors inside batteries is imperative.

Embedding microsensors inside the battery is an effective way to quickly and directly obtain the internal parameters of the battery [33]. Zhu et al. removed a portion of the active material from aluminum foil and attached a multi-point thin-film sensor to monitor the temperature inside the cell in real time at the expense of losing some capacity [34]. FBG sensors enable high-precision measurement of parameters such as temperature and pressure during battery operation [35]. Although FBG sensors are effective in monitoring batteries’ temperature, they are relatively complex to prepare, expensive, prone to bending and vibration, poorly adapt to the battery assembly process, affect electrochemical performance, and require external equipment to analysis, limiting their application scenarios [26,36].

In this study, a novel anode current collector based on flexible printed circuit (FPC) was designed and fabricated. By integrating a PT1000 RTD microsensor on the anode current collector of the flexible printed circuit, a built-in temperature integrated sensor was prepared, and the integrated design of current collection and temperature monitoring was realized. The sensor is capable of rapidly and accurately detecting the internal temperature of a cell, while maintaining consistency with existing assembly processes. It measures about 15 °C temperature difference inside/outside the battery. On short-circuit, the battery’s internal temperature rises to 27 °C in 10 s and 32 °C in 20 s, measured by the sensor. A battery with the PT1000 sensor retains 89.8% capacity under 2 C high currents, similar to the normal battery. Furthermore, a PT1000 temperature array sensor was designed and employed to enable precise monitoring and localization of internal temperature variations.

## 2. Material and Methods

### 2.1. Preparation of Flexible Printed Circuit

**Figure 1:**
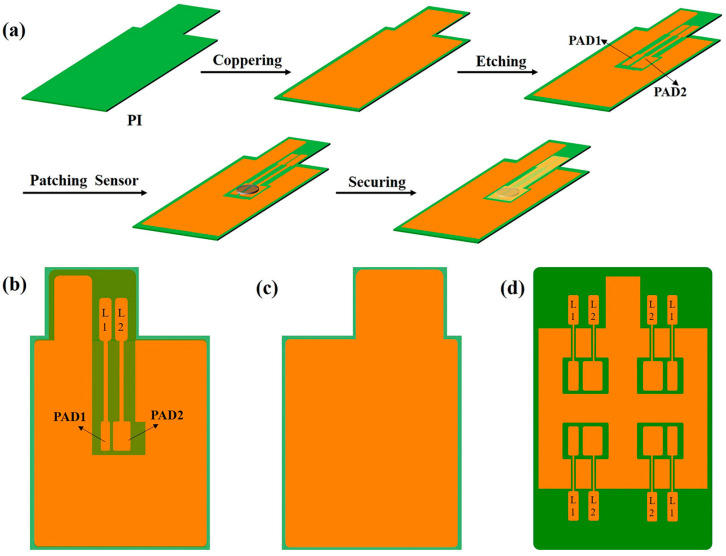
(**a**) Preparation of FPC anode collector, (**b**) top view of FPC current collector, (**c**) the side without etching, (**d**) top view of FPC negative current collector sensing array circuit.

The Flexible Printed Circuit (FPC) preparation process is illustrated in Figure 1a. Both sides of a 20 μm polyimide (PI) substrate were coated with 25 μm thick copper on each side, and two rectangles of different sizes were etched in the center region of the copper foil on one side (PAD1 = 6 × 3 mm, PAD2 = 6 × 6 mm). PAD1, PAD2 as the sensor electrodes are unconnected. The PT1000 RTD sensing element (Heraous, Hanau, Germany) is welded on the sensor electrodes PAD1 and PAD2. Sensor signal lead wires (T1, T2, 27 × 2 mm) are connected to the two sensor electrodes (PAD1 and PAD2), and PI film is used to protect the sensor signal lead wires to avoid the temperature signal of the sensor from interfering with the battery performance. An anode collector with an integrated temperature sensor on one side in Figure 1b and a completely copper backside of the collector (50 × 60 mm) was prepared for coating the anode material of the battery, in Figure 1c. We conducted an additional bending test on the FPC copper anode current collector, after bending 100 times, the PT1000 RTD sensing element did not come off from the FPC copper anode electrode collector, proving that it has good adhesion.

In order to localize the temperature changes inside the battery, an FPC anode current collector sensing array circuit is designed. One set of electrode PADs was increased to four. As shown in Figure 1d, the four pairs of PADs can be implanted with four sensors, which can monitor the signals from the four positions and thus localize the damage. The FPC anode current collector is utilized to make a PT1000 temperature sensor array to localize the temperature changes.

### 2.2. Preparation of Lithium-Ion Batteries with Internally Integrated Sensor

**Figure 2:**
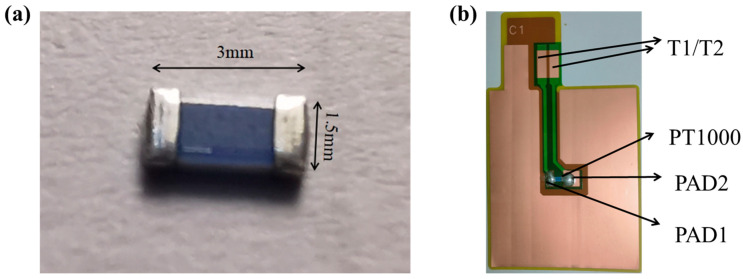
(**a**) Physical diagram of PT1000 (3 mm × 1.5 mm × 600 µm), (**b**) Anode collector with integrated temperature sensor.

The completely copper backside of the collector (Figure 1c) was coated by mixing graphite, carbon black, and carboxymethyl cellulose, styrene-butadiene rubber (ratio of 1:2) binder at a weight ratio of 8:1:1, and baked at 80 °C for 2 h, then baked in a vacuum at 140 °C for 12 h, and the electrode sheet was compacted to a thickness of 80%. Subsequently, the PT1000 RTD sensing element (in Figure 2a) was welded to PAD1 and PAD2 on the opposing side, respectively, and coated with PI adhesive for protection. The thickness of the PT1000 is 600 micrometers (µm), which has a relatively minor impact on the battery’s thickness. Once the PI adhesive had cured, the sensor integrated on the special collector and the anode electrode were successfully prepared in Figure 2b. The composition of the LiCoO_2_ (LCO) electrode was 80% LCO, 10% Super-P, and 10% polyvinylidene fluoride (PVDF), by weight. Then the cathode slurry was coated onto the 6 µm aluminum foil and baked at 80 °C for 2 h, then baked in a vacuum at 140 °C for 12 h, and the electrode sheet was compacted to a thickness of 80%. The cathode was cut into rectangle of 48 mm × 58 mm. The battery was assembled with Celgard 2325 separator and electrolyte (1.0 M LiPF_6_ in EC:EMC = 3:7, with 2.0% VC, Suzhou DoDo, Suzhou, China). A soft-pack li-ion battery integrated with the sensor was obtained by lamination, liquid injection (1 M of LiPF_6_ in 3:7 vol.% ethylene carbonate:ethyl methyl carbonate with 2.0% vinylene carbonate), battery packaging, and other steps.

### 2.3. Preparation of Normal Battery

The graphite, carbon black and carboxymethyl cellulose, and styrene butadiene rubber (ratio of 1:2) binder were mixed in the ratio of 8:1:1 by weight and coated on the copper collector (12 µm) and baked at 80 °C for 2 h, then baked in a vacuum at 140 °C for 12 h, and the electrode sheet was compacted to a thickness of 80%. The anode was cut into a rectangle of 50 mm × 60 mm. LiCoO_2_, super P, and PVDF were mixed in the ratio of 8:1:1 by weight and coated onto the 6 um aluminum foil and baked at 80 °C for 2 h, then baked in a vacuum at 140 °C for 12 h, and the electrode sheet was compacted to a thickness of 80%. The cathode was cut into a rectangle of 48 mm × 58 mm. The cells were assembled from Celgard 2325 diaphragms and electrolyte (1.0 M LiPF6, EC:EMC = 3:7 with 2.0% VC, Suzhou Daodao). Through lamination, liquid injection (1 M LiPF6 in 3:7 vol.% ethylene carbonate:methyl ethyl carbonate and 2.0% vinyl carbonate), cell packing, and other steps, a soft-packed lithium-ion battery integrated with a sensor was obtained.

### 2.4. Sensor Performance Characterization

**Figure 3:**
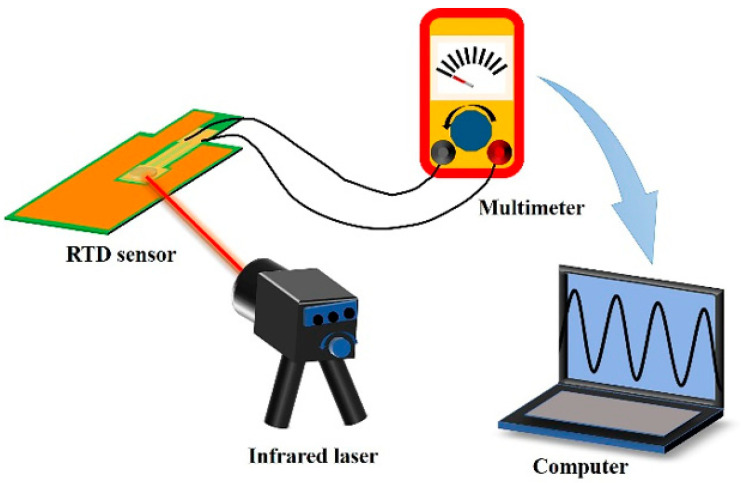
Temperature response performance test platform.

In order to investigate the temperature response performance of the sensors, the test platform was built, as shown in Figure 3. The PT1000 RTD sensor is irradiated by an infrared laser, and a multimeter is used to test the resistance of the T1 and T2 signal lines. The power of the infrared laser is 1 W, and the wavelength is 808 nm. A temperature chamber (Espex, Cardiff, UK) is also used to test and characterize the PT1000 sensor. The chamber’s temperature control range is −70~180 °C. The multimeter is set to resistance to test the resistance of the sensor and analyze the corresponding temperature to evaluate the battery’s safety.

### 2.5. Electrochemical Measurements

Batteries with integrated temperature sensors underwent charge/discharge tests in a Land CT3002AU system at 3.0–4.2 V. Capacity changes at various discharge rates were assessed by charging at 0.5 C and discharging at 0.2 C, 0.5 C, 1 C, and 2 C. Cyclic voltammetry (CV) was conducted using a CHI660D workstation at 0.1 mV/s, scanning from 2 to 4.5 V vs. Li/Li^+^. EIS analysis was performed using an AMETEK-Princeton Versa STAT 3F within 0.01 Hz–100 kHz. Both integrated PT1000 temperature sensor batteries and normal batteries were tested for EIS at open circuit voltage, post-formation, and after completing the rate test.

## 3. Results and Discussion

### 3.1. Sensor Performance Characterization

**Figure 4:**
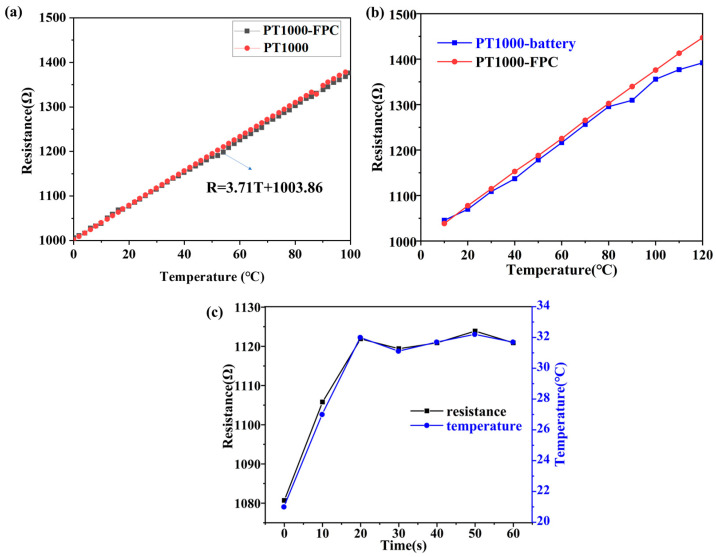
(**a**) PT1000 Sensor on FPC resistance and temperature correlation chart. (**b**) A comparative analysis of the resistance of a PT1000 sensor integrated within a battery and a bare PT1000 sensor at varying temperatures. (**c**) The PT1000 temperature sensor measures the temperature of the battery when it is short-circuited.

After making the PT1000 RTD patch sensing element into a sensor, the resistance changed, so the resistance coefficient of the PT1000 RTD patch sensing element should be corrected. In order to determine the correspondence between temperature and sensor resistance, the PT1000 sensor on FPC is placed in oven, and the temperature is increased from 0 °C to 120 °C. The resistance was recorded at intervals of 2 °C, resulting in a graph that shows the relationship between temperature and the internal resistance of the sensor, as depicted in Figure 4a. Temperature has a good linear relationship with resistance, with the equation R = 3.71T + 1003.86 (R is the resistance value, T is the temperature in Celsius). The error of PT1000 RTD sensing element is ±(0.3 + 0.005 × |t|), t is in degrees Celsius. A comparison of the curves of the PT1000 temperature sensor (PT1000-FPC) and the bare PT1000 resistance thermistor (PT1000) was conducted, and it was found that they basically overlapped. This indicates that the linearity function of the battery-integrated PT1000 temperature sensor and the bare PT1000 are essentially the same in Figure 4a. To further verify the repeatability of these curves, the PT1000 temperature sensor was tested in a thermostat at 20 °C, and a result of 1077 Ω was obtained. This value lies exactly on the curve, which fully verifies the repeatability of the PT1000 temperature sensor.

To monitor the temperature measurement of the PT1000 sensor, the battery with the integrated PT1000 temperature sensor was placed in the oven, and the resistance of the sensor was recorded using a multimeter and converted to the internal temperature of the battery; the test results are shown in Figure 4b. The temperature was increased from 0 °C to 120 °C, and resistance data were recorded at 10 °C intervals (time interval about 1 min). As the temperature rises, the resistance of the PT1000 temperature sensor integrated inside the battery is smaller than that of the bare sensor which is not encapsulated inside the battery. When temperature rises to 120 °C, the resistance of the internal PT1000 temperature sensor battery is 1391.9 Ω, indicating an internal battery temperature of 104.6 °C. For the bare PT1000 temperature sensor heated to about 120 °C, the resistance is 1447.2 Ω, which means that there is an external temperature difference of 15 °C. Batteries are typically made up of multiple layers of materials including electrodes, electrolyte, diaphragm, and external encapsulation materials such as aluminum–plastic film. All of these materials have a certain thermal resistance that prevents heat transfer. When the ambient temperature rises, external heat needs to pass through these layers of material before it can be transferred to the inside of the battery. Due to the thermal resistance, there is a certain amount of heat loss during the transfer process, causing the temperature inside the battery to rise slower than the external environment. As a result, the temperature difference between the inside and outside of the battery gradually increases as the ambient temperature rises. Therefore, if an external temperature sensor is used to detect the temperature of the battery, measurement errors will occur, and the detected temperature will not truly reflect the temperature inside the battery.

This work also designed a short-circuit experiment in which the battery with an internally integrated PT1000 temperature sensor was fully charged at 0.2 C. A wire was used to connect the positive and negative terminals of the battery, and the results are shown in Figure 4c. At room temperature, the resistance of the PT1000 temperature sensor integrated inside the battery was measured to be 1080.7 Ω, corresponding to 21 °C. After short-circuiting, the internal temperature of the battery increased rapidly as shown in Figure 4c, and after 10 s of short-circuiting, the temperature rose to 27 °C; after 20 s, the temperature reached 32 °C. For short-circuit durations of 30 s and longer, the measured resistance of the internal sensor of the battery changed very little, remaining close to that measured after the battery was short-circuited for 20 s, and the temperature fluctuated around 32 °C. This indicates that the battery’s power was almost consumed after being short-circuited for 20 s, and the battery could not continue to warm up. If the capacity of the battery is larger, the temperature of the battery will rise higher and faster after short-circuiting, which can lead to thermal runaway, resulting in battery combustion or even explosion. The short-circuit experiment proved that the sensor can monitor the temperature change inside the battery in real time, ensuring that the battery operates under safe conditions.

**Figure 5:**
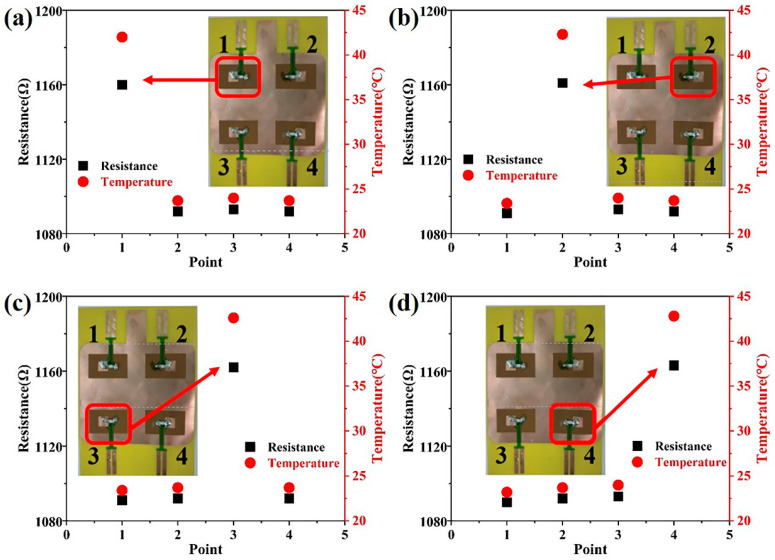
Infrared laser irradiation of PT1000 temperature sensor array for 10 s measured the resistance and temperature of each point. (**a**) Laser irradiation point 1, (**b**) Laser irradiation point 2, (**c**) Laser irradiation point 3, (**d**) Laser irradiation point 4.

The PT1000 temperature sensor array is capable of accurately identifying the location of heat generation within the battery. Given the inherent randomness of heat generation within the battery, we have employed an infrared laser spot heating sensor array to simulate the battery’s heat generation. The laser heating process is as follows: for example, sensor #1 is heated by the laser for 10 s, and then the resistance values of sensors #1, #2, #3, and #4 are immediately read and converted to temperature. After the temperature at the sensor location has dropped to room temperature during the test, a 10 S laser irradiation process is performed on one of the other sensor locations. Figure 5a shows a plot of the resistance measured at the four positions after the laser heats point 1 of the sensor array for 10 s. At room temperature, the measured resistance at the four positions is 1091 Ω (about 24 °C), and when the infrared laser heats point 1 for 10 s, the resistance is measured to be 1160 Ω (corresponding to 42.09 °C), while the resistance of the remaining three points remains unchanged at about 1091 Ω (about 24 °C). Figure 5b–d shows that after the laser heats points 2, 3, and 4 in the sensor array for 10 s, the resistance of the three positions is measured to be about 1162 Ω (corresponding to 42.6 °C), while the resistance of the sensing elements in the other positions does not change. The temperatures in Figure 5 are all calculated based on the resistance of the PT1000 sensor. From this we can conclude that the temperature sensor array can accurately locate and measure the temperature in different areas of the battery.

### 3.2. Electrochemical Performance of Internally Integrated PT1000 Temperature Sensor Array

**Figure 6:**
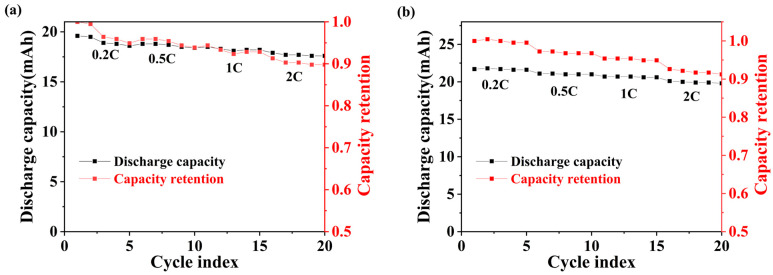
Rate test: (**a**) Battery with internally integrated PT1000 temperature sensor array, (**b**) normal battery.

The battery should be discharged and charged at a rate of 0.2 C to ascertain its actual discharge capacity, which should then be utilized as the benchmark for subsequent rate testing. Figure 6a presents the rate test curve of a battery with an internally integrated PT1000 temperature sensor array. The battery was charged at 0.5 C and discharged at 0.5 C, 1 C, and 2 C, and the capacity retention was 93.88%, 92.96%, and 89.80% with respect to the discharged capacity of 0.2 C, respectively. At a high current discharge of 2 C, the capacity retention of the battery is close to 90%. Figure 6b illustrates the rate test curves of the normal battery, and the discharge capacity retention of the battery when discharged at currents of 0.5, 1, and 2 C is 96.77%, 94.93%, and 91.24% with respect to the discharged capacity of 0.2 C, respectively. A comparative analysis indicates that the battery with the PT1000 temperature sensor array exhibits a capacity retention nearly equivalent to that of conventional batteries when subjected to high currents of 2 C.

**Figure 7:**
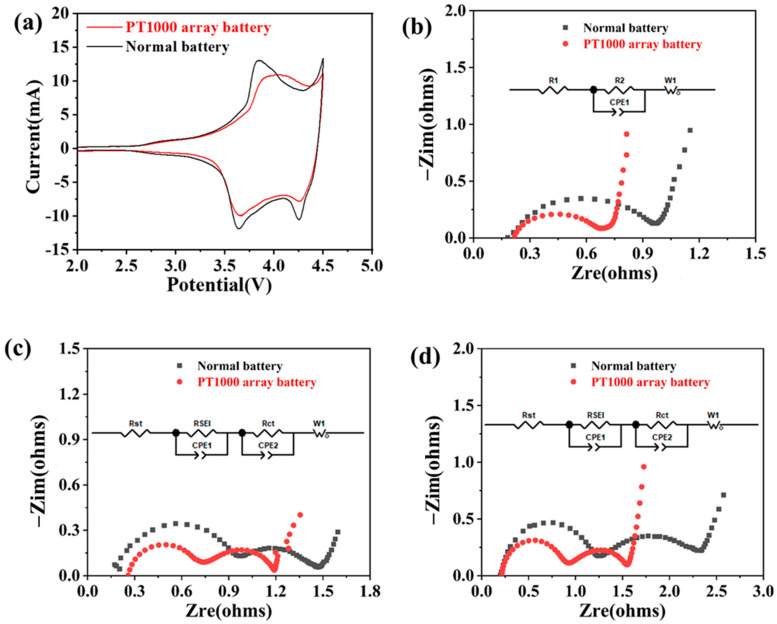
(**a**) CV diagram of normal battery and internally integrated PT1000 array battery, EIS of normal battery and internally integrated PT1000 array battery, (**b**) at open-circuit voltage, (**c**) after formation, and (**d**) after rate test.

In order to investigate the effect of an embedded PT1000 temperature sensor array on the electrochemical side reactions of batteries, CV tests were conducted on the battery, shown in Figure 7a. In the process of lithium removal, the oxidation peaks of the normal battery appeared at the position of 3.83 V, while those of the battery with internally integrated PT1000 temperature sensor array appeared at the position of 4.03 V. In the embedded lithium process, two reduction peaks appear at 3.63 V and 4.25 V for ordinary lithium batteries, and two reduction peaks appear at 3.65 V and 4.27 V for batteries with internally integrated PT1000 temperature sensor array, respectively. Through the analysis of CV curves, it can be obtained that the position of redox peaks of the battery with PT1000 temperature sensor array and normal battery are close to each other, and the electrochemical performance of the two batteries is similar.

An EIS test was performed on a normal battery and an internally integrated PT1000 array battery. Figure 7b shows the Nyquist plot of the cell measured at open circuit, with a semicircular shape in the high-frequency region, which is related to the charge transfer resistance (R_ct_), and a diagonal line in the low-frequency region, which is related to the diffusion process of Li^+^ in the cell (Z_w_). Figure 7c,d shows the Nyquist plots of the cell obtained after formation and rate test, with the semicircular shape in the high frequency region attributed to the formation of a solid electrolyte interface (SEI) film on the anode material after Li^+^ diffusion into the solid–liquid phase interface, the semicircular shape in the mid-frequency region attributed to the charge-transfer resistance (R_ct_), and a diagonal line in the low-frequency region formed by the action of diffusion process (Z_w_).

The experimental data were simulated using ZView software (3.0), and the values of each impedance were obtained according to the equivalent circuit fitting, as shown in Table 1. The ohmic resistances (R_s_) of the integrated PT1000 sensor array battery did not change much at different stages. Because the SEI film has not been formed before the formation, there is only a semicircle in the open-circuit test. The R_SEI_ values of the battery with internally integrated PT1000 temperature sensor are 0.38 Ω after formation. The R_ct_ values of the battery with internally integrated PT1000 temperature fitted at the three stages are 0.26 Ω, 0.54 Ω, and 0.74 Ω. Comparative analysis with the battery impedance of ordinary lithium batteries, each impedance of the battery with internally integrated PT1000 temperature sensor array is relatively small, and the impedance difference with ordinary lithium batteries is not significant, which has little effect on the rate performance of the battery.

## 4. Conclusions

In this study, a novel flexible printed circuit (FPC)-based anode current collector was designed and made. It features an integrated PT1000 RTD microsensor on the anode, enabling simultaneous current collection and temperature monitoring. The temperature of the integrated PT1000 RTD microsensor has a good linear relationship with resistance, with the equation R = 3.71T + 1003.86. The built-in sensor measures a temperature difference of about 15 °C between the inside and outside of the battery. When the battery is short-circuited, the PT1000 temperature sensor detects a rapid increase in the internal temperature of the battery, which rises to 27 °C after 10 s of short-circuit and reaches 32 °C after 20 s. After loading the anode of the integrated PT1000 temperature array sensor into the battery, the battery with the PT1000 temperature sensor array exhibits a capacity retention (89.8%) nearly equivalent to that of conventional batteries when subjected to high currents of 2 C. Furthermore, a PT1000 temperature array sensor was designed and employed to enable precise monitoring and localization of internal temperature variations. Therefore, this method has great potential for improving the safety performance of batteries. In the future, more kinds of battery sensors can be integrated on the flexible printed circuit (FPC) board to prepare multifunctional flexible battery built-in sensors, which can further improve the reliability of the battery.

## Figures and Tables

**Table 1:** Equivalent circuit fitting impedance spectral parameters for integrated PT1000 sensor array battery.

Impedance	R_s_ (Ω)	R_SEI_ (Ω)	R_ct_ (Ω)
After open circuit voltage	0.24	\	0.26
After formation	0.29	0.38	0.54
After rate test	0.23	0.61	0.74

## Data Availability

The data that support the findings of this study are available from the corresponding author (X.P.) upon reasonable request.
